# Editorial Comment to “Short‐Term Longitudinal Changes in Quality of Life Among Japanese Patients With Prostate Cancer After Single‐Port Robot‐Assisted Radical Prostatectomy: A Preliminary Prospective Study”

**DOI:** 10.1111/iju.70278

**Published:** 2025-11-04

**Authors:** Go Kaneko

**Affiliations:** ^1^ Department of Uro‐Oncology Saitama Medical University International Medical Center Saitama Japan

Robot‐assisted radical prostatectomy (RARP) has demonstrated advantages over both open and laparoscopic surgery with respect to cancer control and preservation of sexual function. Due to the superior operability of the robotic platform—three‐dimensional vision, tremor filtration, and wristed instruments—RARP was rapidly adopted worldwide and is now regarded as a standard procedure. To further reduce invasiveness, the da Vinci SP (Intuitive Surgical Inc., California, USA) was developed to enable single‐port access. Over recent years, reports of RARP performed with this platform—designated SP‐RARP—have accumulated, providing evidence of procedural safety, favorable perioperative outcomes, and short‐term improvements in urinary continence and sexual function recovery [1].

Historically, during the laparoscopic era, a subset of surgeons pursued single‐port or reduced‐port laparoscopic radical prostatectomy (LRP) to push the boundaries of minimal invasiveness. Compared with multi‐port LRP, these approaches have been reported to improve cosmesis, reduce postoperative pain, and shorten hospital stay [2]. Yet they also imposed substantial technical constraints. To avoid instrument collisions through a single access site, surgeons relied on specialized maneuvers (e.g., the crossover technique) and unconventional camera handling techniques. The vesicourethral anastomosis, in particular, demanded exceptional suturing dexterity. Consequently, clinical application remained confined to a small cadre of highly skilled laparoscopists, and the techniques gradually fell out of favor as broad reproducibility proved elusive.

Conversely, the da Vinci SP forceps are equipped with a dual‐joint mechanism, comprising an “elbow” and a “wrist,” which enables the forceps to avoid interference. The camera's flexibility facilitates superior visualization, enabling techniques nearly equivalent to multi‐port RARP to be replicated through a single port. Consequently, it possesses the potential to allow a broader range of surgeons, not just those with limited advanced skills, to perform high‐quality procedures, and its widespread adoption is anticipated. It is imperative to assess the impact of RARP on quality of life (QOL), given its potential consequences such as urinary incontinence and sexual dysfunction, and thereby facilitate the widespread adoption of SP‐RARP.

In this issue, Sasaki et al. reported the results of a longitudinal investigation that utilized a series of questionnaires to evaluate changes in QOL up to 6 months following SP‐RARP [3]. While several reports have been published on QOL after SP‐RARP, no previous study has provided a longitudinal evaluation using multiple indicators, making this research highly informative. Although reports indicate a temporary decline in the SF‐8 Physical Component Summary Score early postoperatively with multi‐port RARP [4], this study found no significant decrease in this metric. Moreover, the nerve‐sparing group exhibited significantly higher EPIC sexual function scores at the 6‐month mark compared with the non‐sparing group, thereby suggesting that high‐quality nerve preservation is indeed attainable even with the use of a single‐port approach. Despite the need for additional validation due to the modest sample size of 23 cases, this study provides novel evidence that supports the low invasiveness and high quality of SP‐RARP. As the number of documented instances increases and more compelling evidence emerges, it is anticipated that broader implementation of SP‐RARP will benefit a larger number of patients in the near future.

## Author Contributions

The author takes full responsibility for this article.

## Conflicts of Interest

The author declares no conflicts of interest.
